# Characterization of a Mesoporous Silica Nanoparticle Formulation Loaded with Mitomycin C Lipidic Prodrug (MLP) and In Vitro Comparison with a Clinical-Stage Liposomal Formulation of MLP

**DOI:** 10.3390/pharmaceutics14071483

**Published:** 2022-07-17

**Authors:** Miguel Manzano, Alberto Gabizón, María Vallet-Regí

**Affiliations:** 1 Chemistry in Pharmaceutical Sciences, School of Pharmacy, Universidad Complutense de Madrid, Instituto de Investigación Sanitaria Hospital 12 de Octubre i+12, Plaza de Ramón y Cajal s/n, 28040 Madrid, Spain; mmanzano@ucm.es; 2 Networking Research Center on Bioengineering, Biomaterials and Nanomedicine (CIBER-BBN), 28034 Madrid, Spain; 3 Oncology Institute and Nano-Oncology Research Center, Shaare Zedek Medical Center Affiliated with the Hebrew University School of Medicine, Jerusalem 9112102, Israel; agabizon@szmc.org.il

**Keywords:** nanomedicines, mesoporous silica nanoparticles, liposomal formulation

## Abstract

Nanomedicines have revolutionized the treatment of certain types of cancer, as is the case of doxil, liposomal formulation with doxorubicin encapsulated, in the treatment of certain types of ovarian cancer, AIDS-related Kaposi sarcoma, and multiple myeloma. These nanomedicines can improve the performance of conventional chemotherapeutic treatments, with fewer side effects and better efficiency against cancer. Although liposomes have been used in some formulations, different nanocarriers with better features in terms of stability and adsorption capabilities are being explored. Among the available nanoparticles in the field, mesoporous silica nanoparticles (MSNP) have attracted great attention as drug delivery platforms for the treatment of different diseases. Here, a novel formulation based on MSNP loaded with a potent antitumor prodrug that works in vitro as well as in a clinically evaluated liposomal formulation has been developed. This novel formulation shows excellent prodrug encapsulation efficiency and effective release of the anticancer drug only under certain stimuli typical of tumor environments. This behavior is of capital importance for translating this nanocarrier to the clinic in the near future.

## 1. Introduction

Despite all research and improvements in treatment, cancer is still a leading cause of death worldwide, accounting for nearly 10 million deaths in 2020, according to the World Health Organization. When cancer spreads by forming distant metastases, the disease is incurable except for a few cancer types, and therapy carries a significant burden of toxicity. There are many different options for cancer treatment, including surgery, radiation, therapy, chemotherapy, immunotherapy, biological therapy, and hormone therapy, as single or combined modalities [1]. One of the conventional drugs employed in chemotherapy is mitomycin C (MMC), which was approved by the FDA back in the 1970s [2]. In fact, MMC is used for treating various types of cancer through different ways of administration with the advantage that it is not usually associated with multidrug resistance, a frequent pitfall of many chemotherapeutic agents. MMC is usually the first choice treatment for superficial-bladder cancer by the intravesical route, and it has been included as a key component of many combination therapy protocols for breast, gastric, pancreatic, colorectal, anal, and invasive bladder cancers [3]. However, MMC results in cumulative toxicity to bone marrow and kidneys, which limits its application as a free drug administered into circulation [4].

**Figure 1:**
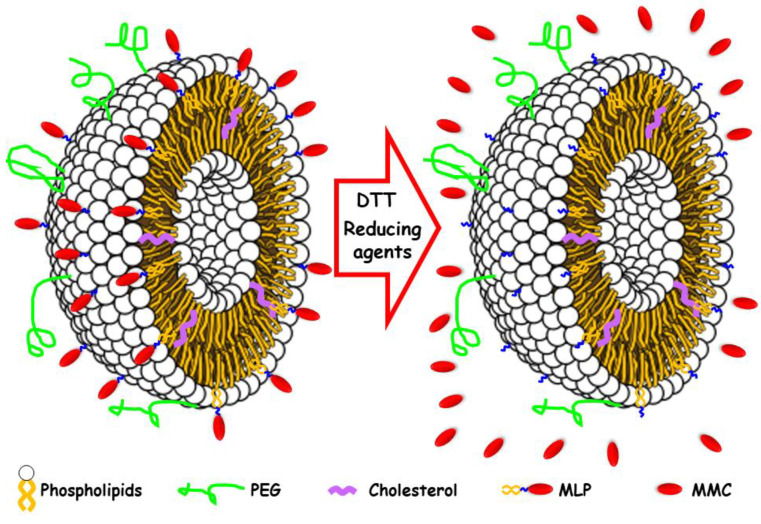
Schematic representation of the Promitil^®^ formulation, a pegylated liposome loaded with the prodrug MLP (**left**); in the presence of reducing agents present in tumor tissues, MMC is released thanks to the cleavage of the disulfide groups (**right**).

A potential solution for avoiding the toxicity of the free drug is the encapsulation of MMC into different carriers, such as liposomes. This approach could reduce its toxicity and enhance its therapeutic potential if MMC were retained stably in liposomes during circulation [5]. However, MMC was quickly released from those nano-liposomal carriers in physiological fluids, which allows the drug to gain non-selective access to tissues, thereby resulting in similar toxicity effects as the free drug. To solve this, Gabizón et al. proposed the synthesis of a mitomycin C lipidic prodrug (MLP) so that the liposomal entrapment of MMC could be facilitated and the formulation stabilized thanks to the strong association of the prodrug to the liposomal bilayer [6]. From the chemical point of view, the prodrug MLP was composed of the actual drug, MMC, modified through a dithiobenzyl linker with a lipid moiety [7]. The idea was that the prodrug (MLP) could be activated into the drug (MMC) only in the enriched thiolytic environments of tumors, where the presence of abundant reducing agents could cleave the dithiobenzyl bridge between the lipophilic moiety and the MMC drug (Figure 1) [8]. The formulation of the prodrug MLP within unilamellar pegylated liposomes is referred to as Promitil^®^ [9], which has resulted in very promising results in terms of pharmacokinetic profile, reduced toxicity, and improved efficacy and, consequently, is being evaluated in clinical trials [10,11,12].

However, liposomal formulations have a major limitation regarding drug encapsulation efficiency when passive loading methods are used. High and stable drug loading in nano-liposomes might be very difficult to reach due to the combination of the very small nano-aqueous volume of the nano-liposomes and/or the poor water solubility of many employed drugs such as the case of many anticancer pharmaceutical agents. This leads to either therapeutic levels that are not enough for treating the disease or the need to administrate large amounts of lipids to reach those therapeutic levels. Additionally, if the loading process is inefficient, there would be a waste of the therapeutic agent and the need for an additional stage for removing the unentrapped drug. Therefore, in some cases, the use of certain liposomal formulations might become inefficient and uneconomical [13].

**Figure 2:**
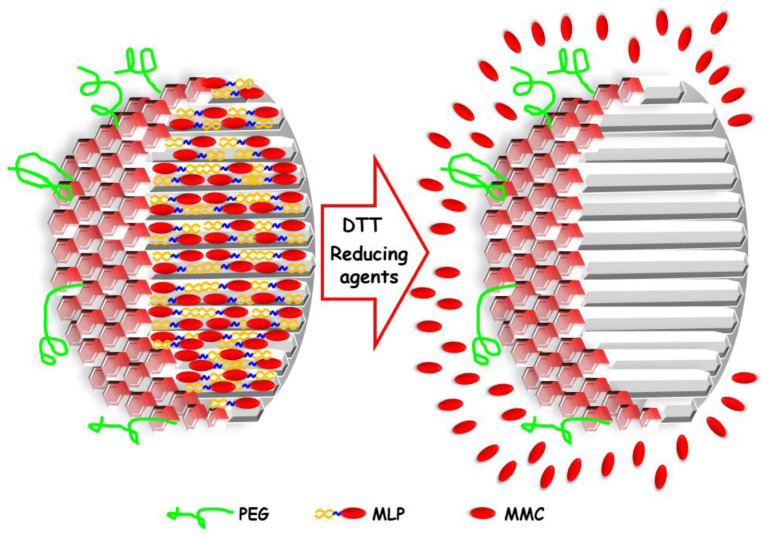
Schematic representation of a cross-section of pegylated MSNPs loaded with the prodrug MLP (**left**); in the presence of reducing agents present in tumor tissues, MMC is released through the cleavage of the disulfide groups (**right**).

While liposomes are probably the most well-known and frequently used drug carrier in nanomedicine, we reasoned it is important to evaluate other nanocarriers with proven stability and great loading efficiency to deliver MLP and compare with liposomes. We chose mesoporous silica nanoparticles (MSNPs). This type of nanoparticle has been proposed as nanocarriers of many different pharmaceutical agents and biomolecules for the potential treatment of several diseases, such as cancer [14,15,16,17], bone infection [18,19], or osteoporosis [20,21], enhancing the efficacy of the treatment [22]. The reasons for the interest in MSNPs rely on their great properties as drug delivery nanosystems, such as high loading capacity, stability, and biocompatibility, among others [23]. For these reasons, in this work, we have explored the use of MSNPs as potential carriers of the antitumoral prodrug MLP that would be activated to MMC only in tumor environments (Figure 2). The results obtained here in terms of entrapment efficiency triggered release under reducing environments, non-toxicity toward healthy cells, and cytotoxicity toward human tumor cells have been compared with those from the well-established Promitil^®^ liposomal formulation. The performance of MLP-loaded MSNPs, which in some cases is superior to Promitil^®^, suggests that, in specific settings, they may be a promising option for the treatment of cancer.

## 2. Materials and Methods

### 2.1. Synthesis of Mesoporous Silica Nanoparticles

**Figure 3:**
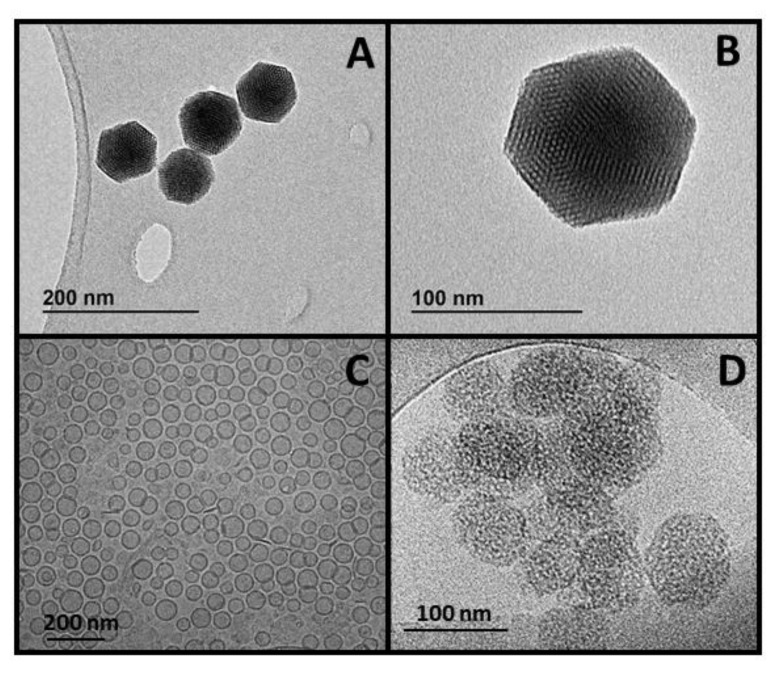
(**A**,**B**) Transmission electron microscopy (TEM) images of mesoporous silica nanoparticles (MSNPs). (**C**) Cryo-TEM image of Promitil (pegylated liposomes with MLP). (**D**) Cryo-TEM image MLP-loaded MSNP.

Monosized mesoporous silica nanoparticles (~90 nm in diameter, Figure 3, ~110 nm in hydrodynamic size in deionized water) were produced following a modification of the conventional Stöber method commonly employed for the production of silica nanoparticles [24,25]. Then, the produced nanoparticles were successively functionalized with aminopropyl triethoxysilane (APTES) and poly(ethylene glycol) (PEG) led to the nanocarriers here employed to transport the prodrug (Figure S1). The synthesis conditions of this type of MSNPs were based on reported literature [26]. Briefly, 290 mg of cetyltrimethylammonium bromide were dissolved in 150 mL of 0.32 M ammonium hydroxide solution in a 250 mL glass beaker, sealed with parafilm, and placed at 50 °C under moderate stirring for 1 h. Then, 3 mL of 0.88 M tetraethyl orthosilicate solution (prepared in ethanol) were added to the surfactant solution and left under magnetic stirring for 1 h at 50 °C. Then, the solution was stored at 50 °C for ~18 h. Then, the solution was pipetted and placed into a glass bottle for a hydrothermal treatment at 70 °C for a further 24 h, and the resulting particles were collected by centrifugation (14,000 rpm, 45 min, 4 °C) and washed three times with ethanol. MSNPs functionalized with amine groups (MSNPs-NH_2_) were produced by placing 150 mg of as-produced MSNPs in a round bottom flask. After drying them at 70 °C for 5 h under vacuum, 5 mL of dry toluene was added, and the particles were dispersed using magnetic stirring/ultrasounds alternatively. In a different vial, 36 μL of 3-aminopropyltriethoxysilane (APTES) (10% *w/w* APTES:MSNPs) were dissolved in 0.5 mL of dry toluene and added to the MSNPs solution under inert atmosphere. The functionalization reaction was kept at 110 °C under an inert atmosphere with magnetic stirring overnight. Then, MSNPs-NH_2_ were collected by centrifugation and washed with ethanol a couple of times.

The procedure carried out for the surfactant removal was based on an ionic exchange using a solution of ammonium nitrate as described in the literature [27]. Briefly, the as-produced particles were placed into 500 mL of a solution of 95% ethanol, 5% water, and 10 mg/mL of ammonium nitrate under magnetic stirring at 80 °C with reflux overnight. This surfactant extraction process was repeated for 3 h, and then the produced MSNPs were centrifuged, washed 3 times with ethanol, and finally stored in pure ethanol.

The as-produced MSNPs were characterized by Transmission Electron Microscopy (TEM) (Figure 3), Fourier transformed infrared spectroscopy (FTIR), thermogravimetric analysis (TGA analysis), Dynamic Light Scattering (DLS), and zeta potential (Figure S2).

### 2.2. Poly(Ethylene Glycol) Grafting to MSNPs-NH_2_

In a typical experiment to PEGylate the as-produced MSNPs-NH_2_, 8 mg of poly(ethylene glycol) 2000 Da (PEG_2000_) (4·10^−6^ mol) with a carboxylic acid group in one end were activated with 2.88 mg of 1-ethyl-3(3-dimethylaminopropyl)carbodiimide (EDC) (14·10^−6^ mol) and 1.62 mg of N-hydroxysuccinimide (NHS) (14·10^−6^ mol) in 2 mL of dry DMF under an inert atmosphere with magnetic stirring at room temperature for 1 h. Then, 40 mg of previously dried MSNPs-NH_2_ were dispersed in 1 mL of dry DMF and added to the PEG_2000_ solution. The reaction medium was stirred at room temperature overnight, and the product was collected by centrifugation and washed with DMF twice and with water 3 times. The material was then dried under vacuum. Different percentages of PEG:MSNPs-NH_2_ were explored as described in Table 1: 10%, 20%, 30%, and 40%.

### 2.3. Prodrug MLP Loading

Different portions (5 mg) of the prepared MSNPs functionalized with different amounts of PEG (MSNP-PEG_10_, MSNP-PEG_20_, MSNP-PEG_30_, MSNP-PEG_40_) were dried under vacuum at 70 °C and dispersed in 1 mL of a solution of ethanol/tert-butyl alcohol (90:10) with magnetic stirring at 70 °C for 15 min. In a separate vial, 0.75 mg of MLP prodrug were dissolved in 1 mL of ethanol/tert-butyl alcohol (90:10), so the theoretical and expected loading percentage was ca. 15% *w/w* (MLP:MSNPs), as it is in the case of Promitil^®^ [6]. Although MSNPs could load more quantity of prodrug, this percentage was selected for comparison with Promitil^®^. The MLP prodrug solution was added to the different MSNP-PEG_n_ solutions, and the mixture was magnetically stirred at 70 °C for 1 h. Then, 10 mL of PBS were added to each loading mixture, and after 30 min of magnetic stirring at 70 °C, the particles were centrifuged (15,000 rpm 1 h, 4 °C), washed with 20 mL of PBS, collected by centrifugation, and dried overnight at 70 °C. The amount of MLP prodrug loaded was measured by TGA analyses, finding the best encapsulation efficiency (EE, ratio of the entrapment drug to the total drug in the system) for MSNP-PEG_40_, which was 84.7%, 82.2%, and 76.6% in three independent loading experiments (Figure S3).

The encapsulation efficiency was also determined by a different independent method, concentrating the MSNP-PEG_40_ dispersion by evaporation and adding MLP at a weight ratio of 5% and gradually exchanging ethanol with buffer. Briefly, 20 mL of dispersion of MSNP-PEG_40_ in ethanol were reduced by evaporation to 5 mL in a hot plate at 80 °C for ca. 6 h. Then, 3 mg of the prodrug MLP were dissolved in that 5 mL of the previously prepared nanoparticle dispersion in ethanol. Then, dialysis was carried out in 80 mL of buffer HEPES 10 mM with 5% dextrose and pH 6.7 at 60 °C for 2 h and then at 45 °C for a further 2 h in 80 mL of buffer. Then, the final volume of MSNPs suspension was found to be 11 mL (buffered suspension probably with some residual ethanol), and the total MLP was found to be 2.49 mg, being the MLP concentration of 0.23 mg/mL. The MLP concentration in the nanoparticle dispersion was calculated by taking a 50 μL of that dispersion after vortexing it and adding to 450 μL of IPA. Then, the absorbance was measured at 360 nm. Therefore, the encapsulation efficiency was found to be 83%, similar EE to in the different above loading methods explored.

### 2.4. Formulation of Pegylated Liposomes with MLP

The liposome formulation of MLP, Promitil^®^, was kindly provided by Liposome Pharmaceuticals (Jerusalem, Israel). For details on the composition and formulation process, see Amitay et al. [6].

### 2.5. HPLC Analysis of MLP and MMC

MLP and MMC in aqueous buffers were determined by a sensitive and convenient high-performance liquid chromatographic (HPLC) method commonly employed for the determination of mitomycin C in human plasma [28]. Briefly, MLP and MMC were determined by reverse phase HPLC with UV detection. Different MLP standards for the calibration curve were prepared in an ethanol/tert-butyl alcohol (90:10) solution and run in a Waters HPLC Alliance System separation module 2695 equipped with a 2696 Waters PhotoDiode Array Detector. The employed column was a C18 Mediterranea Sea 18 Tecknokroma column, 15 × 0.46 cm, 5 μm, in a mobile phase composed of methanol: 2-propanol (70:30) at a flow rate of 1 mL/min at 25 °C with a retention time of 4.4 min and UV detection at 360 nm. Peak areas were quantified, and the standard calibration curve was obtained (Figure S4).

**Figure 4:**
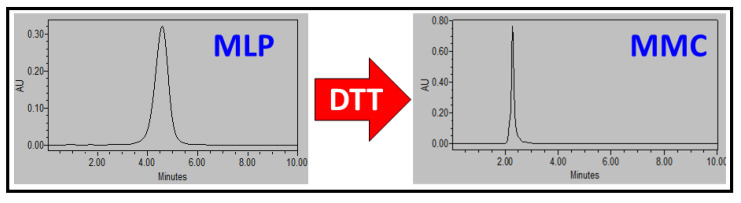
HPLC chromatograms showing the thiolytical conversion of MLP (prodrug, **left**) to MMC (active drug, **right**) after exposure to DTT 0.5 mM for 10 min at 37 °C.

MLP prodrug was activated to MMC using a 0.5 mM dithiothreitol (DTT) solution in ethanol/tert-butyl alcohol (90:10). The mixture was magnetically stirred at 37 °C for 15 min, and then different MMC standards for the calibration curve were prepared and run in a Waters HPLC Alliance System separation module 2695 equipped with a 2696 Waters PhotoDiode Array Detector (Figure 4). The employed column was a C18 Mediterranea Sea 18 Tecknokroma column, 15 × 0.46 cm, 5 μm, in a mobile phase composed of methanol: 2-propanol (70:30) at a flow rate of 1 mL/min at 25 °C with a retention time of 2.2 min and UV detection at 360 nm. Peak areas were quantified, and the standard calibration curve was obtained (Figure S5).

### 2.6. DTT Drug Release Test

**Figure 5:**
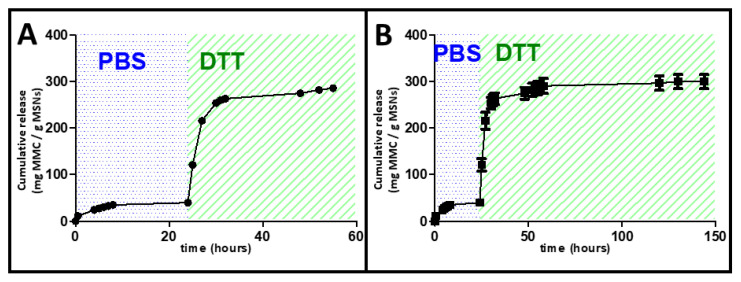
Kinetics of MMC release by DTT; MSNPs-PEG_40_ loaded with MLP were incubated with PBS at 37 °C for 24 h. Then, the release medium was changed to DTT 0.5 mM, and MCC release was obtained after 55 h of experiment (**A**) and 150 h of experiment (**B**).

The release of the drug MCC from MSNPs loaded with the prodrug MLP was demonstrated through incubation of the loaded nanocarriers with DTT. Briefly, a 24-transwell plate was employed to determine the MCC release. The nanocarriers loaded with the prodrug were suspended in PBS pH 7.4 for 24 h, and then the release medium was changed to PBS with DTT as follows. From a 10 mg/mL dispersion of MSNP-PEG_40_ loaded with MLP dispersed in PBS with a pH of 7.4, 0.1 mL were placed on a transwell permeable support (five replications were performed). The well was filled with 0.6 mL of PBS pH 7.4, and the suspension was stirred at 100 rpm at 37 °C during the whole experiment. At every time point studied, the solution outside the transwell insert (0.6 mL) was measured by HLPC (see above) and replaced by fresh PBS pH 7.4. Minimal release of MMC was obtained under continuous shaking at 37 °C, much less than with Promitil^®^. After 24 h, the release medium was changed to PBS with DTT 0.5 mM, and the suspension was stirred at 100 rpm at 37 °C during the rest of the experiment. At every time point studied, the solution outside the transwell insert (0.6 mL) was measured by HLPC (see above) and replaced by fresh PBS with DTT 0.5 mM. The response to the change of release medium can be observed in Figure 5.

**Figure 6:**
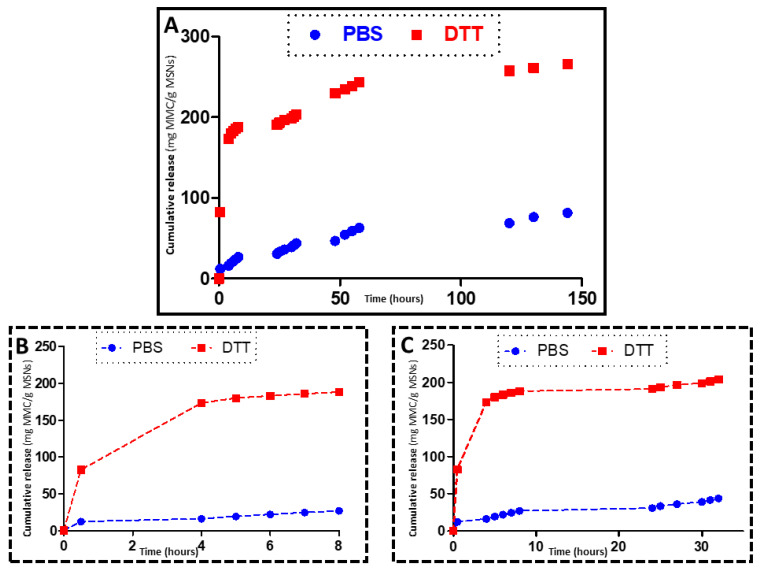
Kinetics of MMC release by DTT; MSNPs-PEG_40_ loaded with MLP were incubated with DTT (activation, red) or PBS (control, blue) at 37 °C, and samples were taken at different periods of time (**A**). Representative curves after 8 h of release experiment (**B**) and after 32 h of release experiments (**C**).

In a separate experiment, the kinetics of the drug release was evaluated through a different experiment. From a 10 mg/mL dispersion of MSNP-PEG_40_ loaded with MLP dispersed in PBS with a pH 7.4 (in the control experiment) or PBS with DTT 0.5 mM (for the prodrug activation), 0.1 mL were placed on a transwell permeable support (five replications were performed). The well was filled with 0.6 mL of PBS pH 7.4 or PBS with DTT 0.5 mM, and the suspension was stirred at 100 rpm at 37 °C during the experiment. At every time point studied, the solution outside the transwell insert (0.6 mL) was measured by HLPC (see above) and replaced by fresh PBS pH 7.4 or PBS with DTT 0.5 mM. The release kinetics can be observed in Figure 6.

### 2.7. In Vitro Cytotoxicity Experiments on Humar Cancer Cells with MLP-Loaded MSNPs

The cytotoxic activity of MSNP-MLP and PL-MLP was monitored in the absence or presence of DTT, the reducing agent, in different human cancer cell lines, including KB (cervix carcinoma), N87 (gastric carcinoma), PANC-1 (pancreatic carcinoma), and T24 (bladder carcinoma). The free drug, MMC (Kyowa, Japan), was used as a control in the in vitro cytotoxicity assays. Since each prodrug molecule of MLP contains one moiety of MMC, we examined the cytotoxic potency of the compounds tested in MMC equivalents using a molar scale to allow a normalized comparison. Cytotoxicity studies were performed on cell monolayers in 96-multi-well plates by continuous exposure for 72 h to free or liposomal drugs at various concentrations in triplicates. Cell growth rates and IC50 values were estimated using a methylene blue colorimetric assay as previously reported [29]. All cell lines were obtained long ago and maintained in our laboratory in deep freeze storage with occasional passages. N87 cells were from the lab of Prof. Yarden (Weismann Institute); KB cells from the lab of Prof. Galski (Hadassah Medical School); and Panc-1 cells were purchased from ATCC.

## 3. Results and Discussion

### 3.1. Nanoparticles Synthesis and Optimization

Mesoporous silica nanoparticles were obtained following a modification of the Stöber method employing a surfactant as a structure directing agent under very dilute conditions at basic pH, obtaining nanoparticles of ca. 90 nm in diameter with mesoporous structure. The MSNPs still containing the surfactant molecules in their mesostructure were functionalized with 3-aminopropyltriethoxysilane (APTES) to ensure the preferential functionalization of the external surface of the nanoparticles. Then, the removal of the surfactant molecules would ensure the availability of the whole pore volume to be filled with the selected cargo to be delivered to its target. The nanoplatform with the –NH_2_ groups on their surface provided by the APTES were successively PEGylated by a condensation reaction with the carboxylic groups from the PEG derivative (Mw 2000 Da) via carbodiimide chemistry [30]. Poly(ethylene glycol) was conjugated to the surface of the MSNPs to improve their colloidal stability in different physiological media, increase their dispersibility in aqueous media and increase their circulation half-life [31]. The correct synthesis of MSNPs and the further modification of their surface with amine groups and PEG were confirmed through several characterization techniques. Transmission electron microscopy (TEM) of the nanoparticles demonstrated the small size and the well-ordered mesoporous structure of the particles, as observed in Figure 3.

The successive functionalization steps represented in Figure S1 were confirmed by several characterization techniques. Among them, Fourier transformed infrared (FTIR) spectroscopy showed the typical vibration bands from SiO_2_ in MSNPs, amine groups in MSNP-NH_2_, and PEG groups in MSNP-PEG, as observed in Figure S2 in the Supporting Information. Briefly, all spectra display a broad vibration band within the 3000–3400 cm^−1^ region due to the O-H stretching vibration bands from the silanol groups (Si-OH). The presence of these groups was confirmed by the presence of the Si-O in-plane stretching vibrations at ca. 950 cm^−1^ and the Si-O stretching vibrations. The dense silica network typical of this type of material was confirmed by the intense Si-O covalent bond vibrations in the range of 1200–1000 cm^−1^ and the Si-O-Si symmetric stretching vibrations at ca. 800 cm^−1^ and its bending vibrations at ca. 470 cm^−1^. The FTIR spectrum of MSNPs functionalized with amine groups (MSNP-NH_2_) showed several bands 2850–2990 cm^−1^ corresponding to the C-H asymmetric and symmetric stretching vibrations of the alkyl chains from the grafted APTES. Additionally, the vibration bands at ca. 3400 cm^−1^ (NH_2_ stretching), 3150 cm^−1^ (N-H stretching), ca. 1530 cm^−1^ (N-H deformation), and ca. 1400 cm^−1^ (C-N stretching) confirmed the correct grafting of aminopropyl groups to the surface of the MSNPs. The grafting of PEG to the surface of MSNP-NH_2_ through a condensation reaction between those NH_2_ groups at the nanoparticle surface and the COOH groups from the PEG derivative was confirmed by FTIR through the presence of the C=O asymmetric stretching vibration bands from the newly amides formed at ca. 1650 cm^−1^. Additionally, the PEGylation was also confirmed by the display of the C-O stretching and CH_2_ rocking vibration bands at 1160 cm^−1^ and the CH_2_ twisting vibrations bands at ca. 1230 cm^−1^ from the newly grafted PEG groups.

The Z-potential measurements of the particles dispersed in water confirmed the successive functionalization steps on their surface since there is a severe change from negative (−29.2 mV) in bare MSNPs to positive (+17.6 mV) in MSNP-NH_2_, as expected [32]. Then, the Z-potential was modified from the previously mentioned positive values to negative values after PEGylation, indicating that the positive charge from the amine groups at the surface was hidden after grafting the PEG groups, in agreement with the literature [33,34,35]. In fact, the value of Z-potential was found to depend on the amount of PEG added, being −24.2, −21.3, −20.6, and −17.6 mV for 40%, 30%, 20%, and 10% PEG:MSNs, respectively. The hydrodynamic size of the nanoparticles was explored through dynamic light scattering (DLS), showing that their monodispersity was unaffected by the successive functionalizing reactions at their surface (Figure S2). All these data confirmed that the surface of the MSNPs was successfully functionalized with PEG moieties (additional details of the characterization can be found in the Supporting Information).

The amount of PEG grafted to the MSNPs surface was optimized to achieve the closest values of prodrug loading than in the case of Promitil^®^, so an accurate comparison between both systems could be carried out. Therefore, different amounts of PEG (10%, 20%, 30%, and 40% in weight with respect to MSNPs) were grafted to the surface of MSNPs to explore their potential influence on the prodrug encapsulation efficiency. Thermal analyses confirmed that increasing the initial amount of PEG added led to a greater weight loss of organic matter, as initially expected (Table 1).

### 3.2. Prodrug Loading

Previously produced MSNPs with different amounts of PEG grafted on their surface were loaded with the prodrug MLP and their loading capacity was determined by thermogravimetric analyses. The results observed in Figure S3 and Table S1 indicated that the closest encapsulation efficiency (EE, ratio of the entrapment drug to the total drug in the system) to the Promitil^®^ system was for MSNP-PEG_40_, which was 84.7%, 82.2%, and 76.6% in three independent loading experiments. Therefore, this 40% of PEG grafting to the surface of MSNPs was selected for the rest of the experiments, so a comparison with Promitil^®^ could be carried out in terms of MLP loading and MCC release and its effects on several cell lines, as described below.

Interestingly, the MLP loading process into MSNPs was carried out through two different and independent approaches, (1) soaking the previously dried MSNPs in a concentrated solution of the prodrug MLP in a mixture of ethanol/tert-butyl alcohol (90:10) and then adding PBS for washing, centrifuge and dry the loaded nanoparticles; and, (2) concentrating the MSNPs dispersion in ethanol by evaporation and adding a solution of MLP prodrug while gradually exchanging ethanol with HEPES buffer solution trough dialysis. In the former method, the amount of MLP prodrug loaded was measured by TGA analyses, finding the encapsulation efficiency to be 84.7%, 82.2%, and 76.6% in three independent loading experiments. In the latter method, the MLP concentration in the nanoparticle dispersion was calculated by adding 50 μL of that dispersion to 450 μL of IPA to then measure the absorbance at 360 nm, finding the encapsulation efficiency to be 83%. Both approaches led to the same encapsulation efficiency, ca. 80%, which confirms the reproducibility of the MSNPs behavior independently of the instrumental method employed for loading the cargo, which might be very relevant for future industrial applications.

### 3.3. In Vial Release

Before exploring the in vitro behavior of our nanoplatform, it was necessary to test whether the MSNPs could load the prodrug MLP, as it did (see above), and release the drug MMC under the correct stimulus. To do so, we have employed a potent dithiol reducing agent, DTT, that we employed in the past for the activation of the MLP prodrug, which we found to be very effective and a reliable tool to evaluate the pharmacologic effects of cleavage [6]. Figure 4 shows the HPLC chromatograms of MLP prodrug that is activated by DTT, which leads to the appearance of the MMC peak after DTT exposure.

The release of the drug MCC from MSNPs loaded with the prodrug MLP was demonstrated through incubation of the loaded nanocarriers with DTT. Before that, the nanocarriers loaded with the prodrug were suspended in PBS pH 7.4 for 24 h, observing no release of MMC, as displayed in Figure 5. This absence of release could be explained by the poor solubility of the prodrug MLP in aqueous media, which impeded the diffusion along the pores of the cargo to the external environment.

However, when the release media was changed from PBS to DTT, there was an activation of the prodrug to the actual antitumor drug MMC, which led to a burst of its release. This behavior could be considered a stimuli-triggered release since the cargo release only takes place under the presence of reducing agents. Taking into account that those reducing agents are normally present in tumor environments, these particles might be very effective in the potential treatment of certain cancers. These MSNPs could control the potential premature release of the cytotoxic drug since there is no release in PBS, but then, once reaching the tumor, which might take place thanks to the well-known enhanced permeability and retention (EPR) effect [36,37], they would release their therapeutic cargo thanks to the reducing agents present in the tumor. In this sense, MSNPs sensitive to redox stimuli have attracted much attention in the last few years because of the high concentration of reductive species found in tumor cells in comparison with healthy cells or the bloodstream, so the drug release would be controlled by taking place only inside those cells [38].

The redox responsiveness of the system was also observed when placing the MSNPs loaded with the prodrug MLP into a solution containing dithiothreitol, and the MMC drug release was evaluated through HPLC (Figure 6).

Figure 6 shows the release profiles of MLP-loaded MSNPs exposed to DTT and to PBS (control), in which a great difference in drug activation and release can be observed. Those materials exposed to DTT display exponential release kinetics typical of these MSNPs, which are normally fitted to a first-order kinetic model [39,40]. It is clear that the nanocarriers show a quick response to the redox stimuli, which leads to the fast activation and release of the MMC anticancer drug in the initial hours of the release experiment. The difference between the release from the DTT exposed nanoparticles and the control (PBS exposed MSNPs) is very significative, which supports our notion that our nanoplatform could work for selective treatment of tumor tissues without affecting healthy cells since the cargo would be released only in reducing environments, i.e., tumor environments.

### 3.4. Cell Viability Experiments

Blank (drug-free) MSNPs were prepared through the same procedure as before to remove ethanol and tested in cell cultures of Panc-1 and T24 cells for 48–72 h. No inhibition of cell growth was found at nanoparticle concentrations in culture medium up to 0.1 mg/mL, indicating that the MSNPs were not toxic per se (data not shown). This is consistent with the general picture emerging from animal studies with MSNP, indicating that they are relatively nontoxic within an extended dose range, as recently reviewed [41]. Factors that may contribute to the lack of toxicity of MSNP in tissue culture are the low amount of pegylated MSNP taken up by non-phagocytic cells and the particle mesoporosity that should accelerate the bioerosion and breakdown processes to small secretable fragments and final dissolution to silicic acid.

### 3.5. In Vitro Cytotoxicity Experiments

Several in vitro cytotoxicity experiments were conducted with MLP-loaded MSNPs on different human tumor cell lines originating from gastric cancer (N87), cervix cancer (KB), and pancreatic cancer (Panc-1). Both MLP-loaded MSNPs and Promitil^®^ displayed low cytotoxicity in vitro since no activation of the MLP prodrug took place in the cell culture media. However, those experiments confirmed the redox-responsive behavior of the MSNPs since DTT was able to activate the MLP-loaded MSNPs in in vitro cultures, although the results obtained were variable depending on the cell type investigated.

#### 3.5.1. N87 Gastric Cancer Cells

**Figure 7:**
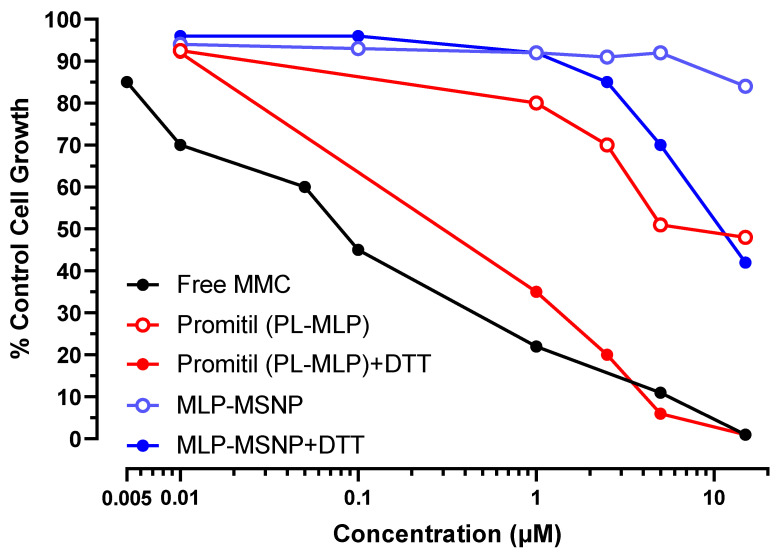
Cytotoxicity evaluation of N87 cancer cells exposed to different concentrations of free MMC, Promitil (MLP-loaded liposomes) dispersed in medium ± DTT (0.5 mM), and MLP-loaded MSNPs dispersed in medium ± DTT (0.5 mM).

N87 cells were used in this experiment, where MSNPs loaded with MLP with and without being exposed to DTT were compared to liposomes loaded with MLP also with and without being exposed to DTT and free MMC as used as control, Figure 7 displays the cell growth respect to the control when increasing concentrations were added to the cells. Although greater cancer cell toxicity was found when MSNPs were exposed to DTT than to DTT-free medium, the cytotoxic effect was still less potent than when Promitil^®^ was activated with DTT.

The half maximal inhibitory concentration (IC_50_) is presented in Table S2 in the Supporting Information. Those values are commonly used as a measure of the potency of a drug since they represent the concentration required to reduce by 50% the growth of tumor cells. The IC_50_ values of Promitil^®^ were reduced from ~7 μM to 0.8 μM when exposed to DTT, while those IC_50_ values of MSNPs were reduced from >15 μM to 9 μM when exposed to DTT. The lowest IC_50_ was that of free drug (0.1 μM).

#### 3.5.2. KB Cervix Cancer Cells

**Figure 8:**
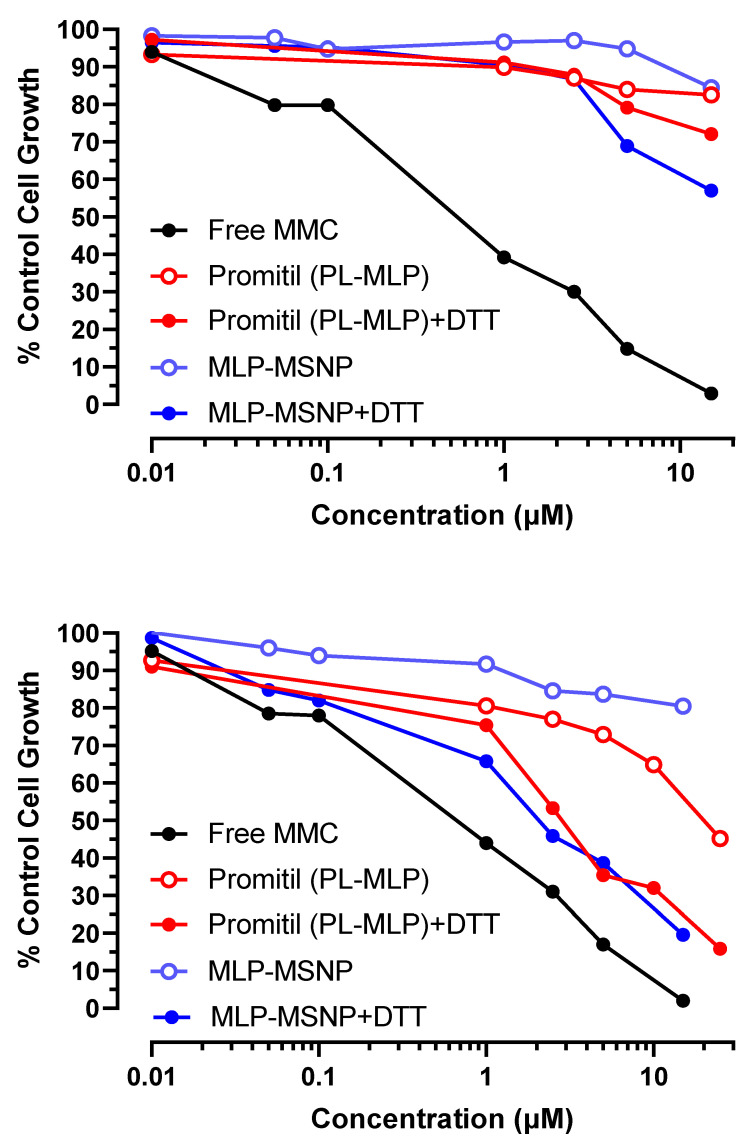
Cytotoxicity evaluation of KB cancer cells exposed to different concentrations of free MMC, Promitil (MLP-loaded liposomes) dispersed in medium ± DTT (0.5 mM), and MLP-loaded MSNPs dispersed in medium ± DTT (0.5 mM) (two independent experiments).

KB cells were tested in two independent experiments. As can be observed in Figure 8, a significant effect of DTT activating the prodrug from MLP-loaded MSNPs was found in both independent experiments, which consequently increased their toxicity against KB cells even more than when those cells were incubated with Promitil^®^ and DTT.

The in vitro toxicity results after 72 h of the test demonstrated the thiolytic activation of the entrapped MLP prodrug in the MSNPs, releasing the cytotoxic agent efficiently, as observed in the drop of the IC_50_ from >15 μM in DTT-free medium to 2.1 μM in the presence of DTT (Table S2).

#### 3.5.3. Panc-1 Pancreatic Cancer Cells

**Figure 9:**
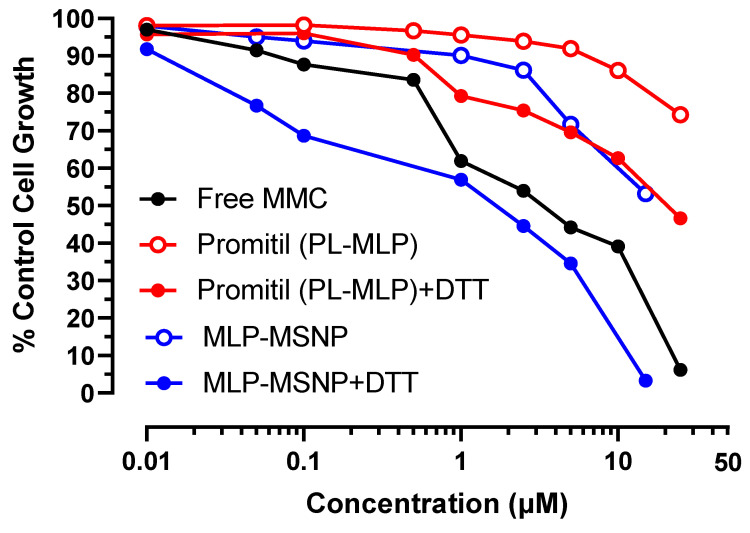
Cytotoxicity evaluation of Panc-1 tumor cells exposed to different concentrations of free MMC, Promitil (MLP-loaded liposomes) dispersed in medium ± DTT (0.5 mM), and MLP-loaded MSNPs dispersed in medium ± DTT (0.5 mM).

Panc-1 cells were employed for conducting the in vitro toxicity experiment with MSNPs loaded with MLP through the examination of the inhibitory activity with and without DTT (Figure 9).

In the case of human pancreatic cancer cells, the MSNPs seem to be even more efficient than Promitil^®^ in the activation of the prodrug and release of the anticancer drug MMC, particularly when exposed to DTT. Moreover, at certain concentrations, the MLP-loaded MSNPs present an in vitro cytotoxicity toward cancer cells similar to the free drug, as can be observed in Figure 9. This means that this platform is able to bring together the anticancer activity of the free drug with the potential benefits of being encapsulated and protected during circulation and transported to the tumor tissue by MSNPs, where activation will take place. As mentioned above and observed in Figure 9 in the Panc-1 cell line, MLP-loaded MSNP ± DTT appears to be more cytotoxic than the liposome formulation ± DTT. Indeed, the IC_50_s of Promitil and MLP-loaded MSNP in the Panc-1 cell line (see Table S2) are significantly different (*p* < 0.001, unpaired *t*-test) from the various formulations. This IC_50_ indicates that the growth of Panc-1 cells is inhibited at 50% with ~2 μM of MSNPs and DTT, while it is necessary for ~13 μM of Promitil^®^ exposed to DTT to reach similar cytotoxicity. One explanation that may account for this observation is that Panc-1 cell uptake of MSNP is greater than uptake of liposomes. This will have to be determined in comparative cell uptake studies.

## 4. Conclusions

In this study, we have developed and characterized a formulation of MSNP loaded with an antitumor lipophilic prodrug and compared its performance in vitro to a well-known and clinically studied liposomal formulation of the same prodrug.

Additionally, the reproducibility of the loading process of the prodrug into MSNPs has been demonstrated through different loading procedures in different labs resulting in consistent encapsulation efficiency in all cases.

The particles developed here were effective in controlling premature drug release experimentally induced by the reducing agent, DTT. When MSNPs were dispersed in PBS, mimicking biological fluids, no drug release took place, while when in the presence of DTT, mimicking a reducing environment typical of several tumor tissues, activation and release of a potent antitumor drug took place. This stimuli-responsive behavior of the present nanoplatform could be very interesting for a future design of personalized nanomedicines able to tackle specific tumor cells without affecting healthy tissues.

Finally, we have demonstrated in several human tumor cell lines the thiolytic activation of the entrapped MLP prodrug, releasing the cytotoxic agent efficiently and reducing the viability of those cancer cells. In some but not all cell lines, MSNPs were more effective than well-known liposomes in vitro, particularly when exposed to DTT with resulting activation and release of anticancer MMC. Some cell lines (Panc-1) are more sensitive to MSNPs loaded with the prodrug than to Promitil, approximating the cytotoxicity of the free drug, which is very promising for a potential fight against pancreatic cancer. These results were obtained at in vitro MSNP concentrations lacking toxicity, thus confirming the validity of MSNP as an effective, relatively nontoxic DDS, which is of paramount importance for future translation of this platform to the clinic. The fact that the investigated MSNPs are not toxic by themselves is of capital importance for potential future applications.

Although significant progress has been made with functionalization and PEGylation of MSNP, further technological improvements may be needed to improve the solubility and prevent aggregation of MSNP suspensions in aqueous media when systemic administration is considered.

## Figures and Tables

**Table 1:** Optimization of the PEGylation process of MSNPs calculated with termogravimetric analyses.

Sample	% PEG:MSNPs (Precursor)	m PEG (mg)	m MSNPs (mg)	% PEG (Final Composition)
MSNP-PEG_10_	10	1.5	15	1.91
MSNP-PEG_20_	20	3	15	2.82
MSNP-PEG_30_	30	4.5	15	2.97
MSNP-PEG_40_	40	6	15	5.19

## Data Availability

Not applicable.

## Supplementary Materials

The following supporting information can be downloaded at: https://www.mdpi.com/article/10.3390/pharmaceutics14071483/s1.
