# Supplementary material for: Characterization of a Mesoporous Silica Nanoparticle Formulation Loaded with Mitomycin C Lipidic Prodrug (MLP) and In Vitro Comparison with a Clinical-Stage Liposomal Formulation of MLP
Source: Pharmaceutics. 2022 Jul 17;14(7):1483. doi: 10.3390/pharmaceutics14071483 (PMC9323893; doi:10.3390/pharmaceutics14071483)
Supplement: Supplementary file 1 [file pharmaceutics-14-01483-s001.zip › pharmaceutics-1815207-supplementary.pdf]

Supporting Information

Characterization of a Mesoporous Silica Nanoparticle formulation loaded with Mitomycin C lipidic prodrug (MLP) and in vitro comparison with a clinical-stage liposomal formulation of MLP

*Miguel Manzano, Alberto Gabizón, María Vallet-Regí **

Monosized mesoporous silica nanoparticles (~90 nm in diameter) were produced following a modification of the conventional Stöber method commonly employed for the production of silica nanoparticles. Then, the produced nanoparticles were successively functionalized with aminopropyl triethoxysilane (APTES) and poly(ethylene glycol) (PEG) led to the nanocarriers here employed to transport the prodrug (**Figure S1**).

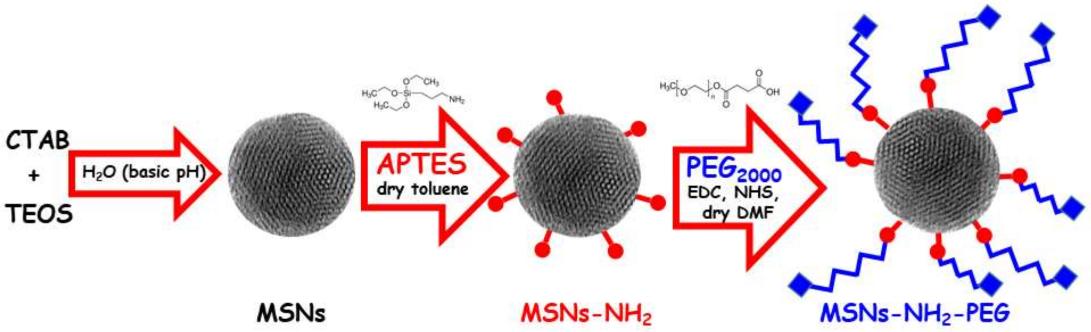

Figure S1. Schematic representation of Mesoporous Silica Nanoparticles (MSNs) synthesis using Cetyltrimethylammonium bromide (CTAB) as the structure directing agent and tetraethyl orthosilicate (TEOS) as the silica precursor. The successive functionalization steps with aminopropyl triethoxysilane (APTES) and poly(ethylene glycol) (PEG) led to the nanocarriers here employed to transport the prodrug MLP and release the activated drug MCC.

The as-produced MSNs were characterized in terms of transmission electron microscopy (TEM), Fourier transformed infrared spectroscopy (FTIR), thermogravimetric analysis (TGA analysis), dynamic light scattering (DLS), and zeta potential (**Figure S2**). FTIR confirmed the presence of the typical silica vibration bands and the successive functionalization steps with $–NH_2$ and PEG groups. Interestingly, the change in the zeta potential after all those modifications confirmed the successive functionalization of the particles, with the typical negative values of zeta potential in unmodified MSNs (-29.2 mV), the highly positive values after grafting the surface with amine groups (+17.6 nM)

and the decrease to negative values again because of the condensation of PEG moieties on the surface of the nanoparticles (-21.9 mV), as expected.

The hydrodynamic diameter of the particles was centered at ca. 90-100 nm measured by DLS, which presented a narrow size distribution in all measurements of the nanoparticles. Interestingly, the successive modifications of the nanoparticle surface did not provoke aggregation of the particles, as noted by the absence of aggregates in the DLS measurements.

Thermogravimetric analyses demonstrated the surfactant removal, amine functionalization, and PEGylation of the produced MSNs.

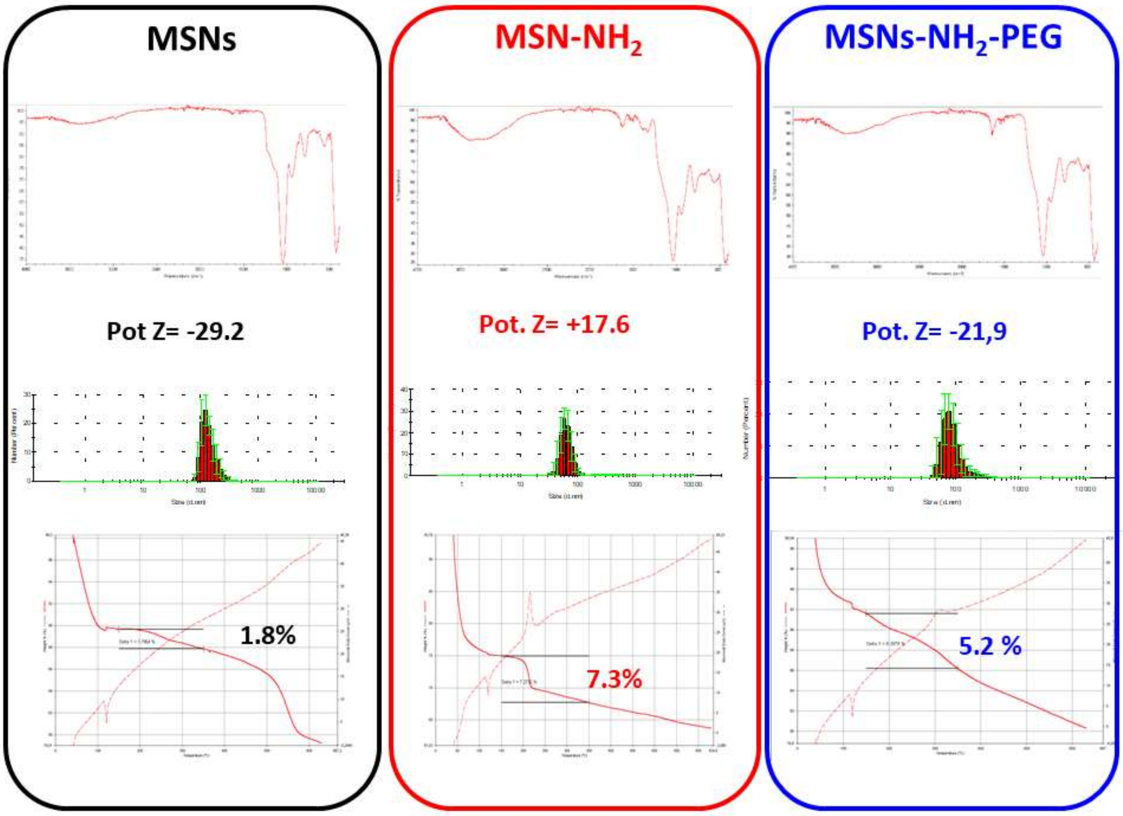

Figure S2. FTIR spectra (top), DLS and Z-potential analyses (middle), and thermogravimetric analyses (bottom) of the as-produced mesoporous silica nanoparticles (MSNs)(left), amine-functionalized nanoparticles (MSNs-NH_2) (center) and PEGylated nanoparticles (MSN-NH_2-PEG)(right).

Thermogravimetric analyses displayed in **Figure S3** showed the characteristic thermogram of as-produced MSNs, the correct surfactant removal from the nanoparticles, together with the appropriate amine functionalization and pegylation of the surface of the nanoparticles, and the loading of the prodrug within the porous cavities of the MSNs.

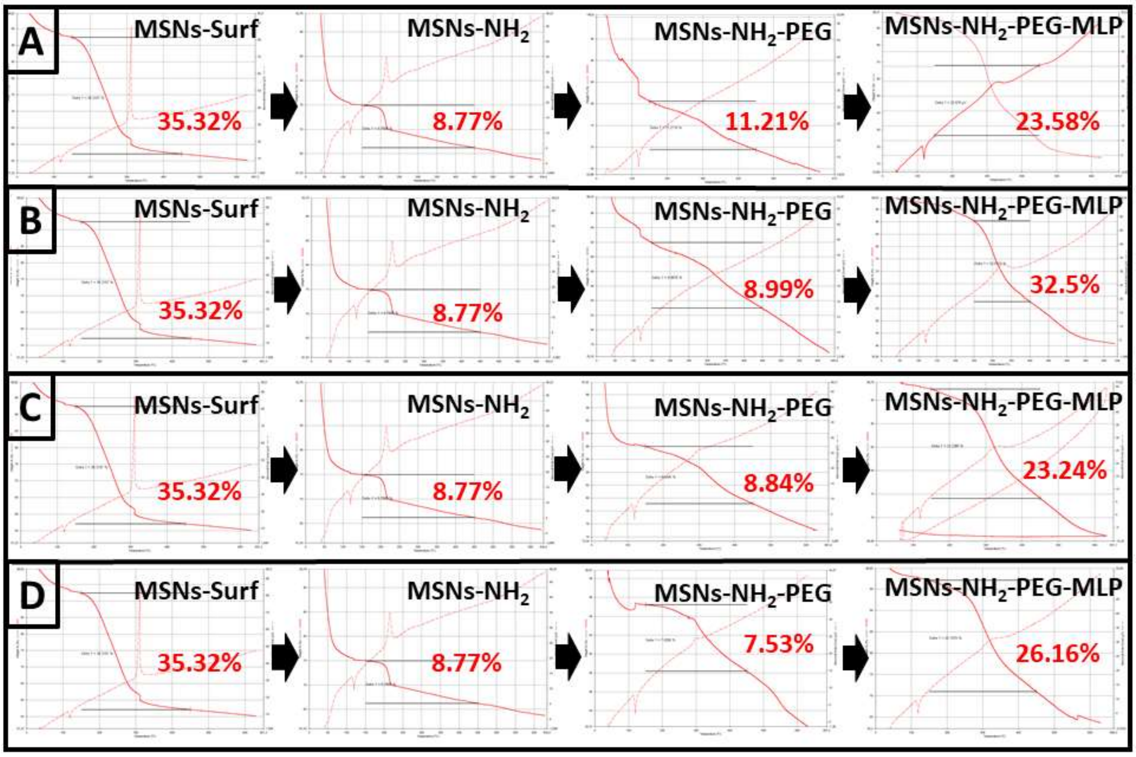

Figure S3. Thermogravimetric analyses of the different functionalization and cargo loading steps during the optimization of the amount of PEG grafted to the surface of the MSNs. (**A**): 40% PEG:MSNs, (**B**): 30% PEG:MSNs, (**C**): 20% PEG:MSNs, (**D**): 10% PEG:MSNs.

Table S1. Optimization of the prodrug (MLP) loading into MSNs functionalized with different amounts of PEG, 10%, 20%, 30%, and 40% of PEG:MSNs.

Sample	% MLP (theoretic loading)	% MLP (experimenal loading)	% Entrapment Efficiency
MSN-PEG_{10}	15	18,6	>100
MSN-PEG_{20}	15	14.4	96
MSN-PEG_{30}	15	23,5	>100
MSN-PEG_{40}	15	12.3	82.2

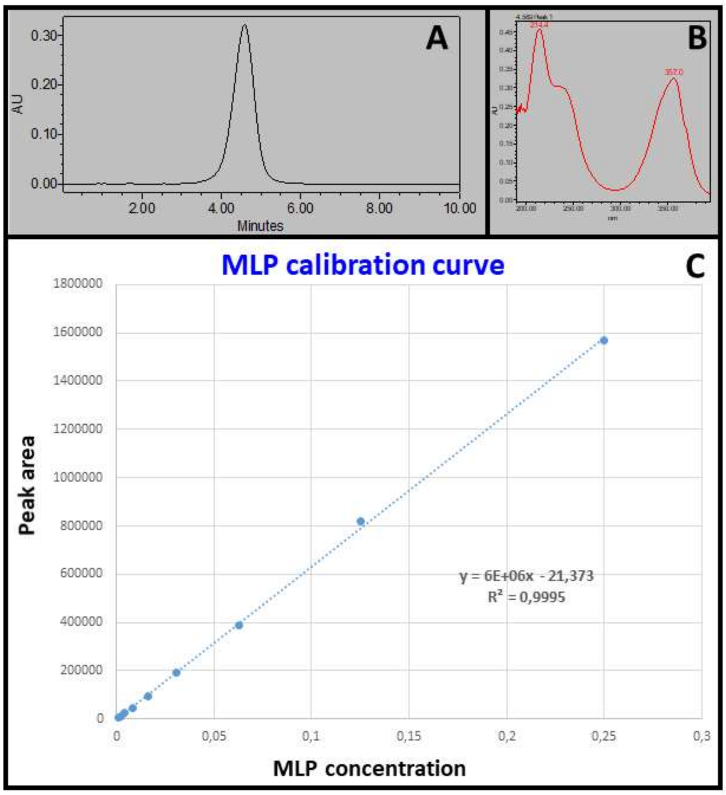

Figure S4. Top: HPLC chromatogram of MLP (**A**) determined by reverse phase HPLV with UV detection using a Waters HPLC system with a C18 column, 15 × 0.46 mm 5 µm using a mobile phase composed of ethanol: 2-propanol (70:30) at a flow rate of 1 mL/min at 25 °C with a retention time of 4.4 min; and UV absorption spectrum of MLP (**B**). Bottom: (**C**) MLP calibration curve quantifying the peak areas of different MLP standards measured with HPLC at 360 nm.

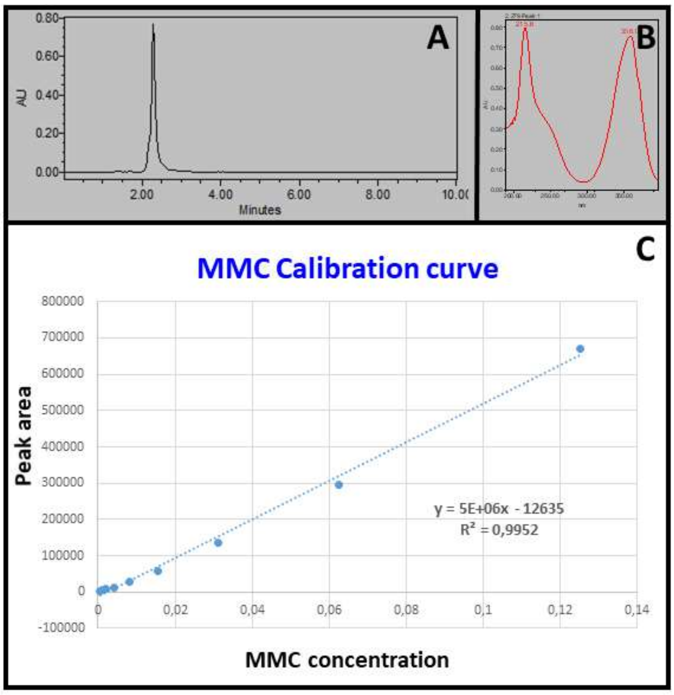

Figure S5. Top: HPLC chromatogram of MMC (**A**) determined by reverse phase HPLV with UV detection using a Waters HPLC system with a C18 column, 15 × 0.46 mm 5 µm using a mobile phase composed of ethanol: 2-propanol (70:30) at a flow rate of 1 mL/min at 25 °C with a retention time of 2.2 min; and UV absorption spectrum of MMC (**B**). Bottom: (**C**) MMC calibration curve quantifying the peak areas of different MMC standards measured with HPLC at 360 nm.

Table S2. IC_{50} values (mean ± SD) of the in vitro toxicity experiments with human gastric cancer cells (N-87 cells), cervix cancer cells (KB cells), and pancreatic cancer cells (Panc-1 cells), respectively.

**IC_{50} (μM) of N87 cells**		
	**Buffer**	**DTT (0.5 mM)**
Free MMC	0.1	
Promitil (liposomes loaded with MLP)	6.9	0.8
MSNPs loaded with MLP	>15	9.1
**IC_{50} (μM) of KB cells ***		
	**Buffer**	**DTT (0.5 mM)**
Free MMC	0.7±0.1	
Promitil (liposomes loaded with MLP)	21.3±1.5	2.9±0.2
MSNP loaded with MLP	>15	2.1±0.1
**IC_{50} (μM) of Panc-1 cells**		
	**Buffer**	**DTT (0.5 mM)**
Free MMC	2.5±0.4	
Promitil (liposomes loaded with MLP)	50.0±1.4	13.2±1.5
MSNP loaded with MLP	15.0±0.3	2.1±0.9

* Based on Expt. A (lower panel), Figure 8.
